# Brain White Matter Impairment in Patients with Spinal Cord Injury

**DOI:** 10.1155/2017/4671607

**Published:** 2017-02-01

**Authors:** Weimin Zheng, Qian Chen, Xin Chen, Lu Wan, Wen Qin, Zhigang Qi, Nan Chen, Kuncheng Li

**Affiliations:** ^1^Department of Radiology, Xuanwu Hospital, Capital Medical University, Beijing, China; ^2^Beijing Key Laboratory of Magnetic Resonance Imaging and Brain Informatics, Beijing, China; ^3^Department of Radiology, Dongfang Hospital, Beijing University of Chinese Medicine, Beijing, China; ^4^Department of Radiology, Tianjin Medical University General Hospital, Tianjin, China

## Abstract

It remains unknown whether spinal cord injury (SCI) could indirectly impair or reshape the white matter (WM) of human brain and whether these changes are correlated with injury severity, duration, or clinical performance. We choose tract-based spatial statistics (TBSS) to investigate the possible changes in whole-brain white matter integrity and their associations with clinical variables in fifteen patients with SCI. Compared with the healthy controls, the patients exhibited significant decreases in WM fractional anisotropy (FA) in the left angular gyrus (AG), right cerebellum (CB), left precentral gyrus (PreCG), left lateral occipital region (LOC), left superior longitudinal fasciculus (SLF), left supramarginal gyrus (SMG), and left postcentral gyrus (PostCG) (*p* < 0.01, TFCE corrected). No significant differences were found in all diffusion indices between the complete and incomplete SCI. However, significantly negative correlation was shown between the increased radial diffusivity (RD) of left AG and total motor scores (uncorrected *p* < 0.05). Our findings provide evidence that SCI can cause not only direct degeneration but also transneuronal degeneration of brain WM, and these changes may be irrespective of the injury severity. The affection of left AG on rehabilitation therapies need to be further researched in the future.

## 1. Introduction

Previous studies on animals and humans have observed brain cortical reorganization following spinal cord injury (SCI). For example, animal models have demonstrated significant anatomical atrophies in the sensorimotor areas following SCI [1–5]. In human studies, some scholars have researched the cortical changes following SCI using voxel-based morphometry (VBM) [6, 7]. Cortical reorganization has been considered an obstacle to sensorimotor function recovery following SCI [8]. Notably, most previous studies have focused on the cortical changes within the SCI [7–13], and the possible changes in white matter (WM) integrity in the brain following SCI have not been fully clarified.

Because the spinal cord contains large numbers of ascending and descending fibres that are directly or indirectly connected to the nuclei and cortices of the brain, SCI will completely or partially destroy these fibre tracts at the injury level. However, it remains unknown whether SCI could indirectly impair or reshape the WM of human brain and whether these changes are correlated with injury severity, duration, or clinical performance. Clarifying these questions will aid the understanding of the mechanisms underlying WM changes in the brain following SCI and possibly contribute to the development of new rehabilitation therapies in the future, including transcranial magnetic stimulation [14–16] and gene chip implantation [17]. To our knowledge, only a few structural studies have explored SCI-related WM changes [8, 18–20]. However, these studies did not clarify whether the changes in the brain WM integrity correlated with injury severity, duration, or clinical performance. Diffusion tensor imaging (DTI) provides unique noninvasive insights into the structural connectivity of the living brain that can help us investigate the microstructure and WM integrity [21]. In the present study, we used tract-based spatial statistics (TBSS) to investigate regional changes in WM integrity after chronic SCI. TBSS is a voxel-wise data-driven method that quantifies the diffusion indices at the centre of the WM tracts (i.e., WM skeleton), which dramatically diminishes the registration problems of diffusion indices, and does not need smoothing before statistics and thus can improve the accuracy and interpretability of group-wise statistics [22]. We hypothesized that WM changes in the brain following SCI would be found in the sensorimotor system. Additionally, we are also interested in the impact of the severity of SCI (i.e., complete SCI (CSCI) versus incomplete SCI (ISCI)) on the integrity of remote brain WM and the correlations between WM fibre tract changes and clinical variables.

## 2. Materials and Methods

### 2.1. Subjects

Fifteen right-handed patients with SCI (10 male and 5 female patients, with a mean age of 46.5 ± 11.2 years and an age range of 28–66 years) were enrolled in this study. Eight patients were labelled grade A, and seven were labelled grades B to D according to the American Spinal Injury Association (ASIA) Impairment Scale 2012 (http://asia-spinalinjury.org). The courses of the diseases ranged from one month to thirty-three years, with a mean of 5.2 ± 8.7 years. All patients had no brain lesions that were confirmed by conventional MRI, and they had never (previously or at present) suffered from traumatic brain injury related symptoms such as loss of consciousness, headache, dizziness, memory loss, attention deficit, depression, or anxiety. All of the patients suffered from bilateral sensorimotor dysfunction, with the exceptions of two patients who exhibited only right- or left-side dysfunction. All of the patients underwent a comprehensive clinical assessment prior to the MR scan; this assessment included a sensory score and motor score that were assessed by a qualified clinician using the ASIA classification scale [23, 24] and visual analogue scale (VAS). The sensory levels were assessed by testing two aspects of sensation, that is, light touch and pinprick sensation (sharp-dull discrimination), at key points in each dermatome (C4–S4-5, bilateral). The motor function assessment involved testing the functions of key muscles in areas corresponding to 10 paired myotomes (C5–T1 and L2–S1). Fifteen age-, gender-, and years of education-matched right-handed healthy volunteers (10 male and 5 female controls with a mean age of 45.0 ± 10.6 years and a range of 26–65 years) were recruited as NCs.  Table 1 provides detailed information about the SCI patients.

The methods were carried out in “accordance” with the approved guidelines, including any relevant details. This study protocol was approved by the Ethics Committee of Xuanwu Hospital, Capital Medical University, Beijing, China. Written informed consent was obtained from each participant in accordance with the Declaration of Helsinki.

### 2.2. Magnetic Resonance Imaging (MRI) Acquisition

All participants were scanned on a 3.0 T Magneton Trio Tim MRI scanner (Siemens Healthcare, Forchheim, Germany). Conventional brain axial fluid-attenuated inverse recovery (FLAIR) and magnetization-prepared rapid acquisition gradient-echo (MPRAGE) sequences (voxel size 1.0 × 1.0 × 1.0* * mm) were acquired prior to the DTI scan to exclude abnormal brains. The DTI experiments were performed using a single-shot gradient-echo echo-planar imaging sequence with the following imaging parameters: TR = 9500 ms, TE = 90 ms, NEX = 1, matrix = 128 × 128, FOV = 256 × 256 mm^2^, nonzero *b* value = 1000 s/mm^2^, gradient directions = 64, slice thickness = 2 mm, and slice gap = 0. A total of 64 contiguous slices parallel to the anterior commissure-posterior commissure line were acquired.

### 2.3. Data Processing and Diffusion Tensor Imaging (DTI)

Postprocessing was performed using TBSS implemented using the FSL 5.0.1 software package (Centre for FMRIB, Oxford University, Oxford, UK; https://fsl.fmrib.ox.ac.uk/fsl/fslwiki/) [22]. The following postprocessing steps were included: all DTI images were visually checked by two experienced radiologists to eliminate images with apparent artefacts caused by, for example, head motion, susceptibility artefacts, or instrument malfunction; eddy current corrections were applied, and motion artefacts were removed using affine alignment. Next, the nonbrain tissues were removed using the brain extract tool (BET), which not only reduces the computation times of the DTI fitting and tracking processes but also improves the accuracy of the spatial registration. The diffusion tensor of each voxel was then fit using a linear least squares algorithm, and the fractional anisotropy (FA), mean diffusivity (MD), axial diffusivity (AD), and radial diffusivity (RD) maps were calculated based on the eigenvalues of diffusion tensors [25]. For the TBSS analysis, the main procedures were as follows: the entire FA dataset was nonlinearly coregistered to the Montreal Neurological Institute (MNI) FA template in the FSL database. Next, a mean FA skeleton from the mean FA images of all of the subjects was derived and represented the centre of the white matter tracts common to the group. An FA threshold of 0.25 [26] was used to involve only the major white matter pathways while eliminating peripheral tracts that are susceptible to misregistration. Finally, each aligned FA map was then projected back onto the skeleton to generate a subject-specific FA skeleton. The processes of nonlinear warping and skeleton projection of the FA maps were also applied to MD, AD, and RD maps.

### 2.4. Statistical Analysis

TBSS using a nonparametric permutation test (5,000 permutations) was performed to compare the FA differences between the SCI patients and the NCs. The permutation test was performed with a fixed-effect general linear model (GLM) with the age and gender as nuisance covariates. Statistical significance was set at *p* < 0.01 and corrected for multiple comparisons using the threshold-free cluster enhancement (TFCE) method. Next, the regions that exhibited alterations in the FA due to SCI were defined as the regions of interest (ROIs), and the mean FA, MD, RD, and AD values of each ROI of each subject were extracted. Two-sample *t*-tests were used to compare the differences in these diffusion indices between the SCI and NC subjects and between the CSCI and ISCI patients (*q* < 0.05, false discovery ratio- [FDR-] corrected) after controlling for age and gender effects. Finally, partial correlation analysis was performed to explore the associations of the clinical variables with the diffusion indices in SCI group, with age and gender serving as nuisance covariates (*p* < 0.05, uncorrected).

## 3. Results

### 3.1. Brain WM Abnormalities in the SCI Patients

**Figure 1:**
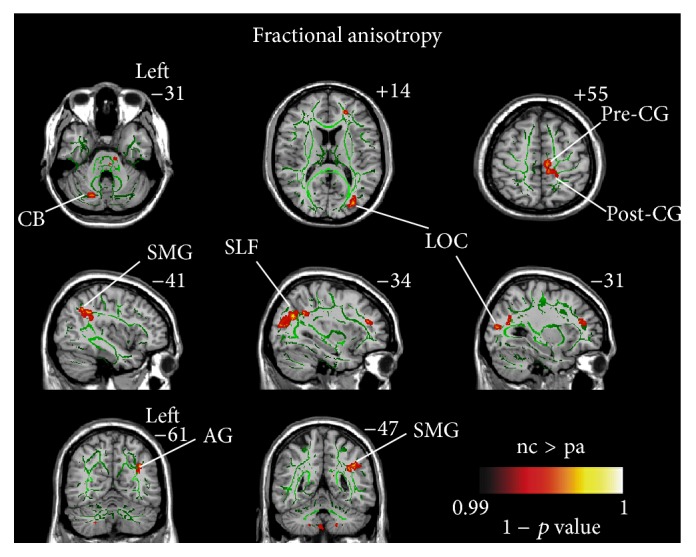
Differences in fractional anisotropy (FA) between the SCI patients and healthy controls based on tract-based spatial statistics (TBSS) (*p* < 0.01, corrected using threshold-free cluster enhancement). Hot color represents 1 − *p* values. It is overlaid on the gyrus skeleton (green) and the MNI 152 template. Significant decreases in FA following SCI occurred in the left angular gyrus (AG), right cerebellar (CB), left precentral gyrus (PreCG), left lateral occipital region (LOC), left superior longitudinal fasciculus (SLF), left supramarginal gyrus (SMG), and left postcentral gyrus (PostCG).

Compared to the normal controls (NCs), significantly lower fractional anisotropy (FA) values were observed in the left angular gyrus (AG), right cerebellum (CB), left precentral gyrus (PreCG), left lateral occipital region (LOC), left superior longitudinal fasciculus (SLF), left supramarginal gyrus (SMG), and left postcentral gyrus (PostCG) in SCI patients (*p* < 0.01, TFCE corrected) (Table 2 and Figure 1).

**Figure 2:**
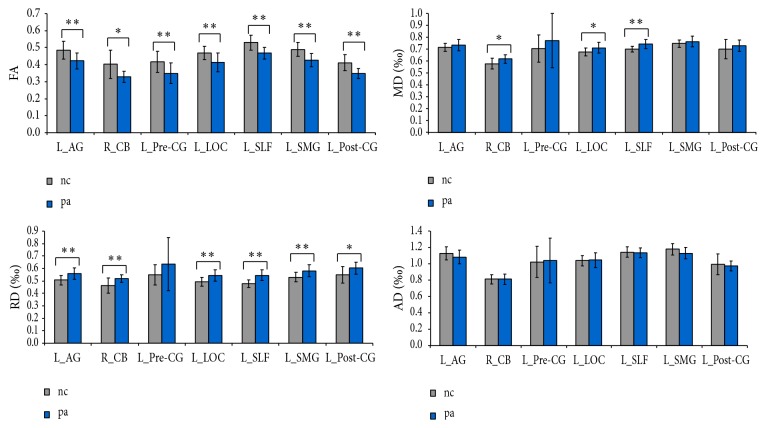
Differences in diffusion metrics between the SCI patients and healthy controls based on region of interest (ROI) analysis. The ROIs were extracted based on the findings of TBSS. *∗∗* represents statistical significance with FDR *q* < 0.05; *∗* represents statistical significance with unadjusted *p* < 0.05. The error bar indicated standard deviation (SD). AG: angular gyrus, PreCG: precentral gyrus, LOC: lateral occipital region, SLF: superior longitudinal fasciculus, SMG: supramarginal gyrus, PostCG: postcentral gyrus, CB: cerebellar, FA: fractional anisotropy, MD: mean diffusivity, RD: radial diffusivity, AD: axial diffusivity.

ROI-wise comparisons generally revealed decreases in the FA and increases in the radial diffusivity (RD) of these brain regions. Significant increases in mean diffusivity (MD) were identified in the right CB, left LOC, and left SLF. In contrast, no significant differences in axial diffusivity (AD) between the SCI patients and NCs were found (*q* < 0.05, corrected using FDR or uncorrected *p* < 0.05; Figure 2). To account for any influence of injury sides on our data, we further investigated the WM changes in SCI patients with bilateral injuries (unilateral injured patients excluded) and observed similar patterns of changes as before (Figure S1, in Supplementary Material available online at https://doi.org/10.1155/2017/4671607), which may suggest that the sides of SCI had little influence on the WM changes in brain. However, because of the relatively small sample size, we cannot directly compare the influence of sides of SCI on the reorganization of the brain, which should be considered in future studies.

### 3.2. Differences in the WM Indices between the CSCI and ISCI Patients

Two-sample *t*-tests revealed no significant differences in the diffusion indices between the CSCI and ISCI patients, with the exceptions of relatively lower MD and lower AD values in the right CB of the CSCI relative to the ISCI patients (*p* < 0.05, uncorrected).

### 3.3. Correlations of the Clinical Variables with the Diffusion Indices in the SCI Patients

**Figure 3:**
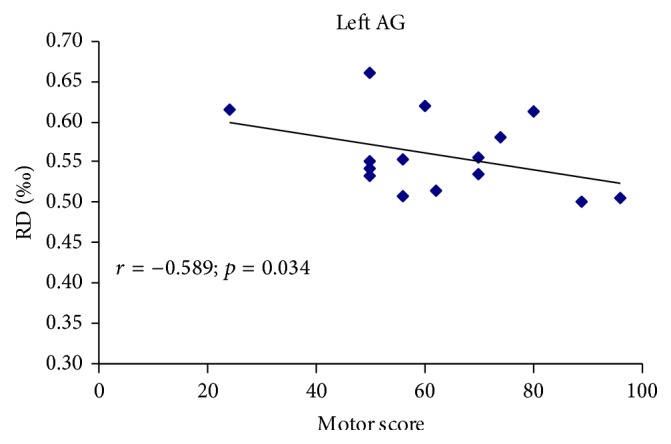
The correlation between diffusion metrics and clinical scores in SCI patients. Pearson correlation showed negative association between the RD of left AG and motor score of ASIA. (*r* = −0.589, *p* = 0.034; uncorrected). RD: radial diffusivity, AG: angular gyrus, ASIA: American Spinal Injury Association, SCI: spinal cord injury.

Partial correlation analyses revealed no correlations between any of the diffusion indices and the injury duration (*p* > 0.05, uncorrected; Supplementary Table S1). A negative correlation was observed between the RD values of the left AG and the motor scores (*r* = −0.589, *p* = 0.034; uncorrected; Figure 3).

## 4. Discussion

In the present study, decreased FA and increased RD were found in the distributed WM of the brain of the SCI patients; these changes occurred not only in the parts of the sensorimotor system that project to the regions of the spinal cord that innervate the paralyzed limbs but also in areas of the brain that are not directly involved in sensation or motor control. Moreover, no significant differences in any of the diffusion indices were found between the CSCI and ISCI patients. Finally, we observed negative correlations between the RD and the clinical scores that indicated an association between the brain WM integrity and clinical performance.

### 4.1. Brain WM Abnormalities in SCI Patients

To our knowledge, only a few studies have addressed the questions of whether and how WM changes occur in patients following SCI [8, 18–20, 27]. Our results were not consistent with those of Wei et al. [20] who failed to find any diffusion changes in the SCI patients without traumatic brain injury, while partially consistent with those of Wrigley et al. [8] and Freund et al. [27] who both detected decreased FA and/or increased MD in the sensorimotor pathway. The contradictory results between Wei et al. and us may be explained by the following factors: First is the difference of method: in Wei et al.'s study, they adopted TBSS as well as the ROI technique because TBSS alone did not find any between-group FA differences. They focused on five ROIs: ALIC, PLIC, forceps minor, gCC, and sCC. In addition, they combined bilateral brain structures into 1 ROI in the cross-subject comparisons (e.g., forceps minor, ALIC, and PLIC). It is possible that both TBI and SCI may cause unilateral changes in cerebral axonal organization or changes in other WM tracts. In the present study, we just used TBSS to assess between-group FA differences and found decreased fractional anisotropy (FA) in the left AG, right CB, left PreCG, left LOC, left SLF, left SMG, and left PostCG. Second, the difference in duration of SCI may be an important factor, because the degeneration processes of the injured ascending and descending fibres tracts are much slower in the central nerve system [28]. The SCI patients recruited in Wei et al.'s study were most subacute (mean injury duration of 93 days), while in the present study and in the studies by Wrigley et al. and Freund et al., most of the patients were chronic (mean injury duration 5.2 years, 12.5 years, and 14.6 years, resp.). Finally, the differences in imaging parameter of DTI might be another factor. The slice thickness of the DTI images by Wei et al. (5 mm) was much thicker than that of the present and other previous studies (below 2.5 mm); thus, partial volume effect might hide some tiny changes in the studies by Wei et al. Beside, the diffusion decoding directions of DTI by Wei et al. (15 directions) were much smaller than the present and other previous studies (30 to 64 directions). Previous studies have shown that higher number of diffusion decoding directions contributes to more robust calculation of diffusion indices [29]. It should be noted that, in the present study, we did not identify significant correlation between diffusion indices and injury duration, which was consistent with the finding by Freund et al. [27] in 2012. However, as we did not give a longitudinal study on the brain WM changes of SCI patients, the exact influence of duration of SCI on the changes of brain WM integrity should be further clarified in the future study.

Our present study demonstrated a significantly decreased FA in the sensorimotor WM that could be partially responsible for the anatomical changes in Somatosensory Cortex Area (S1) and Primary Motor Cortex Area (M1) [18, 30–39]. We also observed that the decrease in FA was primarily attributable to an increased RD rather than a change in the AD. Because RD increases are primarily caused by demyelination [40, 41], our findings are strongly suggestive of demyelination caused by secondary Wallerian degeneration or retrograde degeneration after SCI. Following secondary degeneration, disconnected sensorimotor areas are preserved, but their efferent motor commands do not reach the effectors, and they no longer receive appropriate afferent feedback, leading to severe sensorimotor function deficits [7, 38, 39].

Additionally, significant decrease in FA and increase in RD were observed in the CB. Because the CB has direct and indirect connections with the spinal cord, direct or transneuronal degeneration can explain this finding. The impaired CB WM is approximately located in the cerebellar crus VIII that is related to sensorimotor function. Thus, the degeneration of this CB region may be secondary to the injury of motor-related bundles of spinal cords. We did not find significant correlations between the changes in diffusion indices of CB and clinical sensory or motor measures, indicating that the secondary degeneration of the CB after SCI had little impact on the motor recovery. However, because we did not evaluate the fine motor/sensory skill of SCI patients, we cannot exclude the possible links between CB degeneration and these fine motor/sensory skills.

In addition to direct degeneration, SCI can also lead to transneuronal degeneration, which is related to regions such as the inferior parietal lobule (IPL), SLF, and LOC. The IPL contains AG and SMG and is involved in motor attention [42], motor planning [43], and action coding [44]. The degeneration of the IPL WM may account for the deficits in spatial positioning. SLF is the longest fibre tract among the association fibre bundles. This finding was consistent with that reported in 2013 by Yoon et al. [19]. The SLF connects the frontal lobe, parietal lobe, occipital lobe, and temporal lobe in the brain. Therefore, we hypothesized that the changes in SLF may result at least partially from the destruction of the functional connections between some regions in the brain. However, we did not investigate the changes in functional connection in our patients; therefore, the correlations between the SLP changes and functional connections in the brain cannot be confirmed. In the future, we will investigate this issue. The LOC is responsible for visual conduction. This finding cannot be reasonably explained and needs to be explored in future studies.

### 4.2. Differences in the Brain WM Abnormalities between the CSCI and ISCI Patients

It remains uncertain whether the degree of injury affects the WM changes. Although some studies have found WM changes in either CSCI or ISCI patients, no study has directly compared the potential differences between the two groups within a single study. For example, Villiger et al. [45] found significant white matter atrophy in the brainstem (medulla oblongata) and cerebellum (lobule IX) in ISCI patients, whereas Henderson et al. [18] reported that CSCI patients exhibited significantly reduced FA values in corticospinal tract, corticopontine tract, and superior cerebellum. In the present study, we directly compared the diffusion indices of the brain WM between CSCI and ISCI patients. Unfortunately, we found no significant differences in the diffusion indices between the CSCI and ISCI patients. We can provide the following possible interpretations for this result: (1) the transneuronal degeneration is nonspecific or microspecific in terms of CSCI and ISCI; (2) the sample sizes of each of the SCI subgroups were insufficient to detect the small differences in WM integrity between the CSCI and ISCI patients.

### 4.3. Correlations between the Clinical Variables and Diffusion Tensors in the SCI Group

A few studies have explored the correlations between WM changes and clinical variables and the results were controversial. Hou et al. [7] found no significant correlations between WM changes and clinical performances in SCI patients. However, Freund et al. [13] reported that SCI patients with greater corticospinal tract integrities exhibit better clinical recoveries than those with lower corticospinal tract integrities. In our current study, we found that greater WM integrity (lower RD) in the AG predicted better clinical performance. The AG is involved in motor attention [42], motor planning [43], and action coding [44]. Several previous reports have demonstrated that rehabilitation exercises following SCI can notably influence the structure and function of the brain [38, 46–49]. Thus, this association may indicate the potential of diffusion quantification for evaluating injury severity and predicting prognosis.

As the duration in the present study is heterogeneous, to eliminate its affects on our result, we made partial correlation analyses between the diffusion indices and the injury duration and found no significant differences, which was consistent with Hou et al. [7]. The negative correlation may be affected by the relative small sample size and the heterogeneous injured spinal segments.

## 5. Limitations

Several limitations of the present study should be addressed when interpreting the results. First, the current study investigated WM changes in SCI patients with a very broad range of disease durations. Second, the injured spinal segments were heterogeneous. Finally, the relative small sample size diminished the statistical power, particularly when we considered the CSCI and ISCI patients as separate groups.

## 6. Conclusions

In conclusion, our findings provide evidence that SCI can cause changes in the brain's WM that are not limited to the sensorimotor system, which directly innervates the paralyzed limbs but includes brain areas without such direct connections. Additionally, the changes of the WM integrity in the brain can predict clinical performance. Moreover, the severities of the impairments in the brain's WM are similar between CSCI and ISCI patients. These findings indicate the potential of using diffusion indices in investigations of secondary WM impairments and the prediction of the prognoses of SCI. The affection of left AG on rehabilitation therapies needs to be further researched in the future.

## Supplementary Material

Figure. S1: Differences in diffusion metrics between the SCI patients and healthy controls based on region of interest (ROI) analysis after excluded two unilaterally injured SCI patients. The ROIs were extracted based on the findings of TBSS. ^∗∗^represents statistical significance with FDR *q* < 0.05, ^∗^means represents statistical significance with unadjusted *P* < 0.05. Abbreviations: AG = angular gyrus, PreCG = precentral gyrus, LOC = lateral occipital region, SLF = superior longitudinal fasciculus, SMG = supramarginal gyrus, PostCG = postcentral gyrus, CB = cerebellar, FA = fractional anisotropy, MD = mean diffusivity, RD = radial diffusivity, AD = axial diffusivity. Table S1: Correlations between disease durations and FA, MD values in SCI group.

## Figures and Tables

**Table 1:** Clinical data for the spinal cord injured individuals.

ID	Age [yrs]	Gender	Etiology of the injury	Time since injury [yrs]	Level of lesion^*∗*^	Side of the injury	ASIA^*∗*^	Motor	Sensory^*∗*^	VAS
								(0–100)	(0–224)	
1	55	F	Stab wound	0.75	C3-4	Left	D	89	113	6
2	50	M	Hit by weights	1	C5–7	Bilateral	A	24	80	10
3	34	F	Vehicle accident	1	L1	Bilateral	D	74	190	4
4	38	M	Hit by weights	0.08	T12	Bilateral	A	50	157	4
5	28	F	Fall injury	0.58	L1	Bilateral	D	70	160	0
6	51	M	Vehicle accident	1.33	L1	Bilateral	A	50	84	10
7	55	M	Hit by weights	9	L3	Bilateral	A	50	144	9
8	42	M	Hit by weights	9	T12	Bilateral	A	56	160	9
9	38	M	Hit by weights	7	T12	Bilateral	A	56	144	9
10	40	F	Injury by conveyor	12	L1-2	Bilateral	D	96	148	8
11	66	F	Stab wound	0.17	T8	Bilateral	C	80	172	0
12	52	M	Stab wound	0.25	T10	Bilateral	A	50	168	0
13	60	M	Vehicle accident	3	C3–7	Right	C	70	204	9
14	33	M	Fall injury	0.1	L1	Bilateral	B	62	224	0
15	56	M	Injury by collapse	33	C4	Bilateral	A	60	158	9

^*∗*^The level of lesion refers to the neurological level. ^*∗*^ASIA impairment scale: A, complete, no sensory or motor function is preserved in sacral segments S4-S5; B, incomplete, sensory but not motor function is preserved below the neurological level and extends through sacral segments S4-S5; C, incomplete, motor function is preserved below the neurological level, and more than half of the key muscles below the neurological level have a muscle grade of <3; D, incomplete, motor function is preserved below the neurological level, and at least half of the key muscles below the neurological level have a muscle grade of >3. ^*∗*^Sensory score: sum of segmental light touch and pinprick classifications. ASIA: American Spinal Injury Association. VAS: visual analogue scale.

**Table 2:** White matter regions showing significantly decreased fractional anisotropy in SCI patients.

White matter regions	Peak MNI coordinates			Cluster size (voxels)	Peak *p* value
	*X*	*Y*	*Z*		
L angular gyrus	−35	−61	34	50	0.002
R cerebellar	23	−70	−33	57	0.002
L precentral white matter	−7	−22	55	65	0.002
L lateral occipital	−32	−83	14	107	0.001
L superior longitudinal fasciculus	−33	−62	26	114	0.002
L supramarginal gyrus	−40	−47	33	129	0.005
L postcentral white matter	−11	−34	58	145	0.002
