# Supplementary material for: Brain White Matter Impairment in Patients with Spinal Cord Injury
Source: Neural Plast. 2017 Feb 1;2017:4671607. doi: 10.1155/2017/4671607 (PMC5309430; doi:10.1155/2017/4671607)
Supplement: Supplementary file 1 — Figure. S1: Differences in diffusion metrics between the SCI patients and healthy controls based on region of interest (ROI) analysis after excluded two unilaterally injured SCI patients. The ROIs were extracted based on the findings of TBSS. ∗∗represents statistical significance with FDR q < 0.05, ∗means represents statistical significance with unadjusted P < 0.05. Abbreviations: AG = angular gyrus, PreCG = precentral gyrus, LOC = lateral occipital region, SLF = superior longitudinal fasciculus, SMG = supramarginal gyrus, PostCG = postcentral gyrus, CB = cerebellar, FA = fractional anisotropy, MD = mean diffusivity, RD = radial diffusivity, AD = axial diffusivity. Table S1: Correlations between disease durations and FA, MD values in SCI group. [file 4671607.f1.pdf]

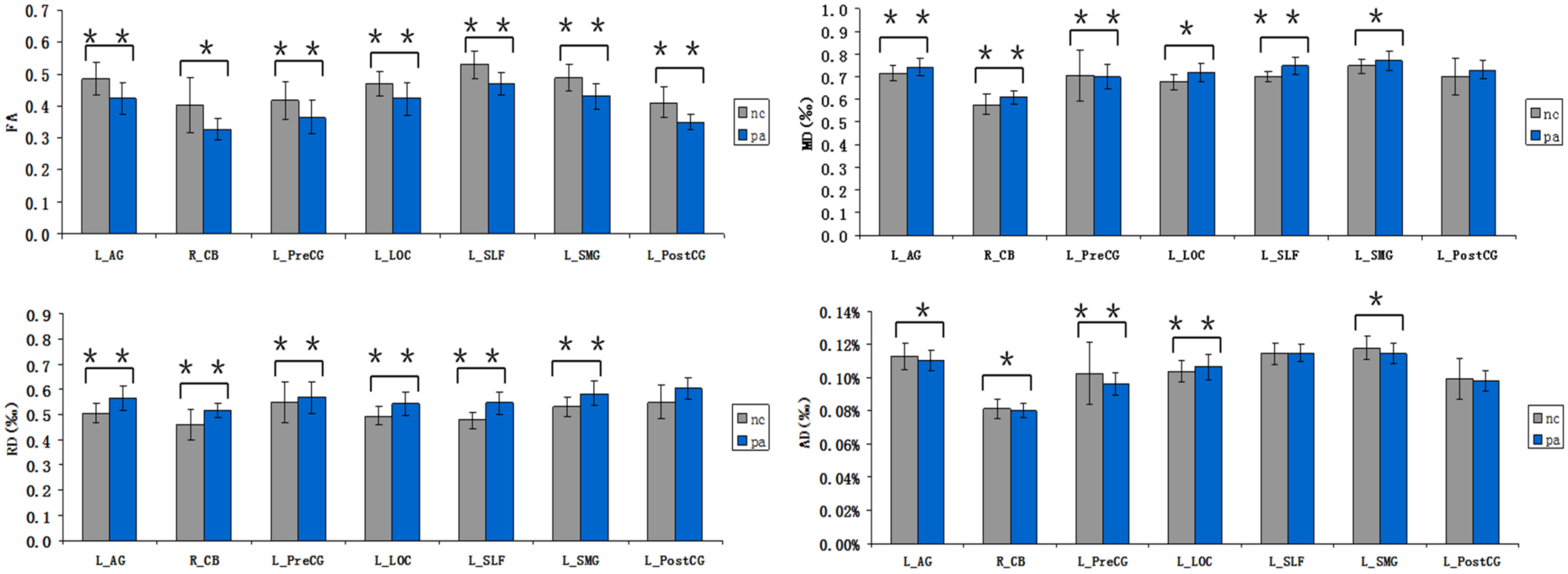

Fig. S1 Differences in diffusion metrics between the SCI patients and healthy controls based on region of interest (ROI) analysis after excluded two unilaterally injured SCI patients. The ROIs were extracted based on the findings of TBSS. ** represents statistical significance with FDR q<0.05, *means represents statistical significance with unadjusted P<0.05. Abbreviations: AG=angular gyrus, PreCG=precentral gyrus, LOC=lateral occipital region, SLF=superior longitudinal fasciculus, SMG=supramarginal gyrus, PostCG=postcentral gyrus, CB=cerebellar, FA=fractional anisotropy, MD=mean diffusivity, RD=radial diffusivity, AD=axial diffusivity.

Table S1: Correlations between disease durations and FA, MD values in SCI group

Control variables	Clinical variable		FA							RD						
			L_AG	R_CB	L_PreCG	L_LOC	L_SLF	L_SMG	L_PostCG	L_AG	R_CB	L_PreCG	L_LOC	L_SLF	L_SMG	L_PostCG
Age&gender	Disease durations	correlation	-0.070	-0.240	0.383	0.136	0.167	0.170	0.474	0.066	-0.132	-0.236	-0.234	-0.276	-0.035	-0.256
		p	0.821	0.429	0.197	0.658	0.585	0.579	0.101	0.829	0.667	0.438	0.441	0.361	0.908	0.399
		df	11	11	11	11	11	11	11	11	11	11	11	11	11	11

Notes: The correlation between diffusion metrics and disease durations in SCI patients. Pearson correlation showed no correlation between all the diffusion indices and injury duration (P>0.05, uncorrected). Abbreviations: L: left; R: right; AG: angular gyrus; CB: cerebella; PreCG: precentral gyrus; LOC: lateral occipital region; SLF: superior longitudinal fasciculus; SMG: supramarginal gyrus; PostCG: postcentral gyrus; FA: fractional anisotropy; RD: radial diffusivity; SCI: spinal cord injury.
